# Is prison food really food?

**DOI:** 10.1186/s40352-023-00244-7

**Published:** 2023-10-25

**Authors:** Stephen J. Schoenthaler, Alan C. Logan

**Affiliations:** 1 Department of Sociology, College of the Arts, Humanities & Social Sciences, California State University, Stanislaus, Turlock, CA 95202 USA; 2 Research Fellow, Nova Institute for Health, 1407 Fleet St, Baltimore, MD 21231 USA

To the Editor,

We read the recent article ‘Food and the prison environment’ (Woods-Brown et al., 2023) with interest. The authors should be commended for synthesizing international research to strengthen their argument that the prison food environment, including food quality and culinary skills, has the potential to improve health within institutions, and beyond. We are hopeful that their efforts will be widely disseminated and used as a roadmap for further research.

Woods-Brown and colleagues provide background references to nutritional quality, wherein quality is mostly determined by the level of macronutrients (e.g., high fat and/or sugar) and the presence/absence of micronutrients. We would like to extend that discourse and point to the rapidly growing research on ultra-processed foods, which we consider to be of high-level relevance to food justice. Ultra-processed foods aren’t really foods (van Tulleken, 2023); they are better described as nutritional products that have been subjected to high levels of industrial processing; some contain added vitamins and minerals, which makes them “nutritionally adequate” on paper (Monteiro, 2009a). That is, ultra-processed foods are capable of passing muster in nutritional analysis within institutional settings, including correctional systems, but mostly they are assembled combinations of sugar, fat, sodium, plant isolates, extruded meat remnants, synthetic emulsifiers, flavor enhancers, and colors (Monteiro, 2009b, 2011).

The NOVA food classification system has allowed researchers to identify ultra-processed foods within dietary patterns; over the last decade, ultra-processed food consumption has been linked to various non-communicable diseases, and more recently, to mental disorders (Samuthpongtorn et al., 2023) and antisocial behavior (Gketsios et al., 2023). At the same time, advances in neuropsychiatry and microbiome sciences have provided support for mechanistic pathways linking ultra-processed food consumption to depression, anxiety, and aggression (Contreras-Rodriguez et al., 2022; Song et al., 2023; Gulledge et al., 2023). Taken as a whole, and when combined with a growing number of intervention studies showing that removal of ultra-processed foodstuffs enhances mental wellbeing (Lane et al., 2023), the available evidence suggests the definition of ‘nutritionally adequate’ in prison systems requires re-evaluation. We also need to consider the extent to which ultra-processed foods are ultimately contributing to an unhealthy environment for all persons in correctional facilities, including personnel.

Awareness of the emerging science of ultra-processed foods, including their dominance in correctional food service (Soble et al., 2020), allows the health and justice field to visualize research opportunities. For example, under the direction of the state Commissioner of Corrections, Maine has engaged with Brigaid, a chef-run organization that helps institutions limit ultra-processed foods. The quality and service of the Maine prison food program has been reported, subjectively, to be “head and shoulders” above other US states (Cebula, 2023). Yet, we need rigorous measurements of success in order to encourage widespread transformation of food systems. What defines head and shoulders above? We argue that measuring outcomes related to mental health and behavior vis à vis the presence or absence of ultra-processed foods, which is to say, food-like products, will aid in the transformation of the prison food environment (Logan & Schoenthaler, 2023).

Respectfully,

## Data Availability

Not Applicable.
